# The Role of Oxidative Stress, Selected Metals, and Parameters of the Immune System in Male Fertility

**DOI:** 10.1155/2018/6249536

**Published:** 2018-09-04

**Authors:** Michał Dobrakowski, Zbigniew Kaletka, Anna Machoń-Grecka, Sławomir Kasperczyk, Stanisław Horak, Ewa Birkner, Jolanta Zalejska-Fiolka, Aleksandra Kasperczyk

**Affiliations:** ^1^Dept. of Biochemistry, School of Medicine with the Division of Dentistry, Medical University of Silesia, ul. Jordana 19, 41-808 Zabrze, Poland; ^2^Department and Clinic of Urology, School of Medicine with the Division of Dentistry in Zabrze, Medical University of Silesia, ul. 3 Maja 13-15, 41-800 Zabrze, Poland; ^3^I-st Chair and Clin. of Gynecology, Obstetrics and Gynecological Oncology, School of Medicine with the Division of Dentistry, Medical University of Silesia, ul. Batorego 15, 41-902 Bytom, Poland

## Abstract

The aim of the study was to investigate the associations between standard semen parameters and the parameters reflecting oxidative stress intensity, antioxidant defense functions, levels of selected macro and trace elements, and parameters characterizing immune system function. The study group consisted of 103 fertile males. Based on semen volume, sperm concentration, total sperm count, and percentage of motile sperm at 1 hour postcollection, the individuals were divided into two equal groups—those with excellent (EX) semen quality and those with mediocre (ME) semen quality. The remaining measured parameters characterizing motility and the percentage of normal morphology were higher in the EX group than in the ME group; however, the seminal plasma pH did not differ between the examined groups. The phosphate level was 31% lower in the EX group than in the ME group, whereas there was a tendency toward a 25% lower level of Fe in the EX group than in the ME group (*p* = 0.064). The activities of enzymes involved in antioxidant defense, CuZn-SOD, CAT, and G6PD, were 14%, 63%, and 39%, respectively, higher in the EX group than in the ME group. However, the level of alpha-tocopherol was 32% lower in the EX group than in the ME group. The other measured parameters characterizing antioxidant defense and the parameters of oxidative stress intensity and immune system function were not significantly different. The value of seminal plasma pH is not related to the semen quality of fertile males. Higher fertility potential estimated based on standard semen parameters in fertile males is associated with lower levels of Fe and higher activities of some antioxidant enzymes.

## 1. Introduction

Infertility is among the most serious medical problems worldwide. Approximately 80 million people worldwide are affected by infertility. Male factor problems account for up to 40–50% of these cases, and there has been a considerable increase in male infertility in recent decades [1–3].

Generally accepted parameters that reflect the functional ability of spermatozoa include sperm count, motility, and sperm morphology. Most men diagnosed with infertility have low sperm numbers in the semen or an adequate number of sperm with reduced sperm motility or/and abnormal morphology. However, men with normal semen tests may also have impaired sperm-fertilizing ability. Conversely, men with poor sperm characteristics may have no problem achieving fertilization. These facts clearly indicate that standard semen parameters are not sufficient to unambiguously determine male fertility status. To introduce more highly reliable tests of male fertility, there is a need to better understand the factors influencing semen quality [1].

A significant part of male factor infertility cases is believed to be due to the damaging effects of oxidative stress. Oxidative stress is an imbalance between reactive oxygen species (ROS) production and the semen's natural antioxidant defenses. The increased levels of ROS may be due to many factors, such as infections, autoimmunity, chronic diseases, high temperatures, electromagnetic radiation, pesticides and pollutants, and lifestyle factors (e.g., advanced age, alcohol consumption, smoking, stress, obesity, and poor diet) [2]. Several studies have documented the interactions between cytokines and ROS. On the one hand, ROS can promote the expression and production of cytokines; on the other hand, some cytokines can modulate prooxidant and antioxidant systems and generation of ROS. In light of this, seminal cytokines via interaction with oxidative stress could be involved in various pathologic conditions related to defective sperm function [4, 5].

Apart from oxidative stress, macro and trace elements, such as calcium (Ca), magnesium (Mg), zinc (Zn), iron (Fe), copper (Cu), and selenium (Se), exhibit significant effects on male fertility [6]. Ca triggers the acrosome reaction and is involved in sperm motility. Mg acts as an intracellular Ca antagonist and plays a role in sperm motility and in spermatogenesis [6, 7]. Zn acts as a cofactor for most enzymatic reactions, including for some involving sperm motility [6, 8]. Fe and Cu also serve as important cofactors participating in oxygenation, reduction processes, and antioxidant metabolism [9]. Whereas Zn and Cu are cofactors of superoxide dismutase (Cu/Zn-SOD) [10], Se is incorporated into glutathione peroxidases (GPxs), a family of antioxidant selenoproteins found in the spermatozoa midpiece [11]. The protective effects of Se are necessary for testicular development, spermatogenesis, and spermatozoa motility [12].

In light of the aforementioned information, the aim of the present study was to investigate the associations between standard semen parameters characterizing semen quality and the parameters reflecting oxidative stress intensity, antioxidant defense function, levels of selected macro and trace elements, and parameters characterizing immune system function.

## 2. Materials and Methods

### 2.1. Study Population

The study group consisted of 103 males who had attended the andrology clinic for the diagnosis of infertility. The inclusion criteria were defined as follows: normal semen profile according to the WHO criteria [13] and no history of drug consumption (including antioxidant medications). The exclusion criteria were defined as follows: smoking, alcohol abuse, a history of any chronic disease (e.g., diabetes), coronary artery disease, or a malignant neoplasm.

The study group was divided into two equal groups according to semen quality. Study subjects were classified to the first group (excellent semen quality—EX group) based on the following criteria: (1) semen volume > 2 ml, (2) sperm cell count in 1 ml > 32 mln/ml, (3) total sperm cell count > 80 mln, and (4) motile sperm 1 hour after collection. Study subjects were classified to the second group (mediocre semen quality—ME group) based on the following criteria: semen volume between 1.5 and 2 ml, sperm cell count in 1 ml between 15 and 32 mln/ml, total sperm cell count between 39 and 80 mln, and motile sperm 1 hour after collection.

The experimental setup has been approved by the Bioethics Committee of the Medical University of Silesia in Katowice (KNW/0022/KB1/I/13/09).

### 2.2. Sample Collection

Semen was collected on the same day in the morning before the first meal. Semen samples (2–6 ml) were collected by masturbation, at home or at laboratory research facilities, after at least 3 days of sexual abstinence (number of days elapsed since last ejaculation was recorded for each volunteer).

### 2.3. Semen Analysis

We followed the methods of Kasperczyk et al. [14]. All of the semen specimens were analyzed according to WHO standards [13], including the assessment of seminal volume, sperm cell density, total sperm cell count, motility, and supravital eosin staining (for the percentage of live spermatozoa). Sperm morphology was examined after Papanicolaou staining. The semen samples (1.5 ml) after liquefaction were centrifuged at 6000 g for 10 minutes to separate the spermatozoa from the seminal plasma. The seminal plasma was transferred to fresh tubes and stored at −75°C until being required for the biochemical and lead analyses. Additionally, a 10% spermatozoa lysate in bidistilled water was made.

### 2.4. Determination of Magnesium

We followed the methods of Kasperczyk et al. [14]. The seminal plasma magnesium was determined using a Unicam flame atomic absorption spectrometer. This method involves diluting seminal plasma with a 0.2% solution of cesium chloride (CsCl), followed by determining the magnesium in solution by measuring the absorbance at a wavelength of 285.2 nm. The concentrations are expressed as mg/dl.

### 2.5. Determination of Calcium

We followed the methods of Kasperczyk et al. [14]. The seminal plasma calcium concentration was determined using a biochemical analyzer method based on a reaction with o-cresolphthalein at pH > 7. The color intensity is directly proportional to the calcium concentration, which was expressed in mg/dl.

### 2.6. Determination of Iron

We followed the methods of Kasperczyk et al. [14]. The seminal plasma iron concentration was determined photometrically. In this method, ascorbate reduces Fe^3+^ ions to Fe^2+^ ions, which react with ferrozine to form a colored complex. The color intensity is directly proportional to the iron concentration, which is expressed in *μ*g/dl.

### 2.7. Determination of Zinc

We followed the methods of Kasperczyk et al. [14]. The concentration of seminal plasma zinc was measured by atomic absorption spectrophotometry using Unicam 929 and 939OZ Atomic Absorption Spectrometers with GF90 and GF90Z, respectively, at a wavelength of 213 nm [15]. The data are shown in mg/l.

### 2.8. Determination of Copper

We followed the methods of Kasperczyk et al. [14]. The concentration of seminal plasma copper was measured by atomic absorption spectrophotometry using Unicam 929 and 939OZ Atomic Absorption Spectrometers with GF90 and GF90Z, respectively, at a wavelength of 324.8 nm [15]. The data are shown in *μ*g/dl.

### 2.9. Determination of Selenium

We followed the methods of Kasperczyk et al. [14]. The concentration of Se in seminal plasma was determined by a flameless method using Unicam 929 and 939OZ spectrophotometers. The calibration curve was prepared using Nycomed® standards. Certified Nycomed® controls (containing 78.0 and 11.4 *μ*g/dm^3^ of Se) were used to perform the internal control. The data are shown in *μ*g/dl.

### 2.10. Determination of Lead

Concentration of lead in seminal plasma (PbS) was measured using graphite furnace atomic absorption spectrophotometry Unicam 929 and 939OZ Atomic Absorption Spectrometers with GF90 and GF90Z. The concentration of lead in the semen specimens was calculated from a standard curve. Data are shown in *μ*g/dl.

### 2.11. Determination of Phosphates

Inorganic phosphorus with ammonium molybdate reacts in the presence of sulfuric acid to form a complex: ammonium phosphomolybdate (NH_3_) [PO_4_ (MoO_3_)_12_]. The concentration of the complex formed is measured photometrically in an ultraviolet spectrum at a wavelength of 340 nm.

### 2.12. Determination of Protein

We followed the methods of Kasperczyk et al. [14]. The protein level was measured by means of an A25 biochemistry analyzer (BioSystems S.A., Barcelona, Spain) according to the manufacturer's instructions. The results for protein levels are expressed in g/l.

### 2.13. Determination of Malondialdehyde (MDA)

MDA, a product of lipid peroxidation, was measured fluorometrically as a 2-thiobarbituric acid-reactive substance (TBARS) in seminal plasma according to Ohkawa et al. [16] with modifications according to Kasperczyk et al. [14]. Concentrations are given in *μ*mol/l.

### 2.14. Determination of Lipofuscin (LPS)

The LPS concentration was determined in seminal plasma according to Jain [17]. Values are presented as relative units (relative fluorescence lipid extract, RF), where X corresponds to a fluorescence solution of 0.1 mg/ml quinidine sulfate (Sigma-Aldrich) in 0.1 N sulfuric acid (POCH).

### 2.15. Determination of Total Oxidation Status (TOS)

The total oxidant status was measured in seminal plasma according to Erel [18]. This method was conducted in an automated analyzer (PerkinElmer) calibrated with hydrogen peroxide. Data are shown in *μ*mol/l.

### 2.16. Determination of Superoxide Dismutase (SOD) Activity

We followed the methods of Kasperczyk et al. [14]. The method of Oyanagui [19] was used to measure the activity of SOD in seminal plasma. The activity of SOD is equal to 1 nitric unit (NU) when it inhibits nitric ion production by 50%. Activities of SOD in seminal plasma were expressed in NU/mg protein.

### 2.17. Determination of Thiol Groups

The concentration of thiol groups was determined as described by Koster et al. [20] using 5,5′-dithiobis-(2-nitrobenzoic acid) (DTNB), which undergoes reduction by compounds containing the sulfhydryl groups, yielding the yellow anion derivative, 5-thio-2-nitrobenzoate, which absorbs light at a wavelength of 412 nm. This assay was carried out using an automated analyzer (PerkinElmer). The results were expressed as *μ*mol per g of protein (*μ*mol/g protein).

### 2.18. Determination of Catalase (CAT) Activity

The catalase (CAT) was measured by using the method of Johansson and Borg [21]. Catalase activity was expressed as international units per milligram of protein (U/g protein).

### 2.19. Determination of Glucose-6-phosphare Dehydrogenase (G6PD) and Glutathione Reductase (GR) Activity

We followed the methods of Kasperczyk et al. [14]. The activities of glucose-6-phosphare dehydrogenase (G6PD) and glutathione reductase (GR) in seminal plasma were measured according to Richterich [22]. G6PD and GR activities were expressed as international units per milligram of protein (U/g protein).

### 2.20. Determination of Glutathione Peroxidase (GPx) and Glutathione-S-transferase (GST) Activity

We followed the methods of Kasperczyk et al. [14]. The seminal plasma glutathione peroxidase (GPx) activity was measured by the kinetic method of Paglia and Valentine [23]. The GPx activity was expressed as international units per milligram of protein (U/g protein). The activity of seminal plasma glutathione-S-transferase (GST) was measured according to the kinetic method of Habig and Jakoby [24]. The GST activity was expressed as international units per milligram of protein (U/g protein).

### 2.21. Determination of Total Antioxidant Capacity (TAC)

Total antioxidant capacity was measured in serum according to Erel [25]. This assay was conducted in an automated PerkinElmer analyzer calibrated with Trolox. Data were expressed as mmol/l.

### 2.22. Determination of *α*-Tocopherol

The concentration of *α*-tocopherol in seminal plasma was determined by Shearer and Lim [26]. Concentrations were provided as *μ*g/ml.

### 2.23. Determination of Cytokines

Levels of interleukin 1*β* (IL-1*β*), interleukin 2 (IL-2), interleukin 4 (IL-4), interleukin 5 (IL-5), interleukin 6 (IL-6), interleukin 7 (IL-7), interleukin 8 (IL-8), interleukin 10 (IL-10), interleukin 12 (p70), interleukin 13 (IL-13), interleukin 17 (IL-17), granulocyte colony-stimulating factor (G-CSF), granulocyte-macrophage colony-stimulating factor (GM-CSF), interferon gamma (IFN-*γ*), monocyte chemoattractant protein-1 (MCP-1), macrophage inflammatory protein 1-*α* (MIP-1*α*), and tumor necrosis factor *α* (TNF-*α*) were detected in seminal plasma using a Bio-Plex 200 System (Bio-Rad Laboratories Inc., Berkeley, California, USA) according to the manufacturer's instruction. The results were expressed as pg/ml.

### 2.24. Statistical Analysis

A database was created in MS Excel 2007. Statistical analysis was performed using Statistica 10.0 PL software. Data were reported as the mean and standard deviation (SD) for parameters with a normal distribution and median and interquartile range (IQR) for those with a nonnormal distribution. Shapiro-Wilk's test was used to verify normality, and Levene's test was used to verify the homogeneity of variances. Statistical comparisons between groups were made using a *t*-test, a *t*-test with a separate variance, or the Mann-Whitney *U* test (nonparametric test). A value of *p* < 0.05 was considered to be significant.

## 3. Results

The mean age in the examined groups did not differ significantly. Semen volume, sperm concentration in 1 ml, total sperm count, and the percentage of motile sperm after 1 hour were higher in the EX group than in the ME group due to the division criteria. Other parameters characterizing motility and the percentage of normal sperm morphology were also higher in the EX group. However, seminal plasma pH did not differ between the examined groups (Table 1).

The phosphate level in semen was significantly lower in the EX group than in the ME group by 31%. There was a tendency toward a 25% lower level of Fe in the EX group than in the ME group (*p* = 0.064). However, the concentrations of the other examined metals (Ca, Mg, Zn, Cu, Se, and Pb) in seminal plasma were not significantly different between the examined groups (Table 2).

The activities of enzymes involved in antioxidant defense, CuZn-SOD, CAT, and G6PD, were 14%, 63%, and 39%, respectively, higher in the EX group than in the ME group. There was a tendency toward a 13% higher activity of total SOD in the EX group than in the ME group (*p* = 0.059). However, the level of alpha-tocopherol was significantly lower in the EX group than in the ME group by 32%. Other measured parameters characterizing antioxidant defense and parameters of oxidative stress intensity were not significantly different (Table 3). Similarly, the parameters of immune system function were the same in both groups (Table 4).

## 4. Discussion

The normal pH of seminal plasma is between 7.2 and 8.0, depending on the length of time since ejaculation. As a result of carbon dioxide loss, the pH tends to increase shortly after ejaculation [27]. In the present study, significantly different values in the motility, sperm volume and count, and the percentage of sperm with normal morphology observed were accompanied with almost equal pH in two study groups due to the study division criteria. This observation is consistent with the results obtained by Banjoko and Adeseolu [27] who examined two groups of males divided based on sperm motility. Banjoko and Adeseolu [27] reported no significant differences in the seminal plasma pH between these groups. These results support the hypothesis that seminal plasma pH does not have an effect on sperm quality except when the pH levels are excessively abnormal. In contrast to pH, the percentage of normal sperm forms is significantly positively associated with the motility parameters due to the impaired motility of the abnormal forms. Consistently, in the aforementioned study by Banjoko and Adeseolu [27], the percentage of abnormal forms was significantly lower in the males with normal motility.

The most important seminal plasma buffers are HCO_3_/CO_2_, proteins, and low-molecular weight components, such as citrate, pyruvate, and inorganic phosphate [27]. The inorganic phosphate level was significantly lower in the EX group than in the ME group; however, the observed difference in phosphate levels and its contribution to seminal plasma buffering capacity seemed to be too low to influence seminal plasma pH. However, the results of the present study indicate a significant association between the inorganic phosphate level and standard semen parameters. This association probably results from phosphate ion being crucial to the activities of adenyl cyclase, the primary regulator of sperm motility, and prostatic acid phosphatase (PAP), which has been associated with the liquefaction process of semen [27]. Based on the results of the present study and previous studies [27, 28], we speculate that there is an optimal inorganic phosphate level and any deviation in this level may negatively affect semen quality.

Our previous investigations indicate that the levels of essential metals, such as Ca, Mg, and Cu, as well as Pb, a common xenobiotic metal, generally are not associated with standard semen quality parameters in fertile males [29–32]. The levels of the aforementioned metals were not different between the EX and ME groups, confirming the hypothesis that their influence on semen quality in the group of fertile males is limited. Similarly, the levels of Zn and Se were also not different between the examined groups. By contrast, there was a tendency toward a lower level of Fe in the EX group than in the ME group. These results are supported by our previous work on fertile males [33] indicating that higher levels of Fe are associated with decreased sperm motility and elevated TOS values in seminal plasma of fertile males. On one hand, Fe plays a critical role in the synthesis of nucleic acids and proteins, electron transport, cellular respiration, proliferation, and differentiation. All of these processes are intimately related to spermatogenesis and spermatozoa metabolism. On the other hand, as a transition metal, Fe can easily donate an electron during oxidation, becoming a source of ROS. Therefore, both Fe deficiency and overload could be harmful to spermatozoa. Oxidative stress related to Fe overload causes damage to lipids, proteins, and DNA, impairing spermatogenesis and spermatozoa metabolism [9]. The results of the present study indicate that the Fe status plays a significant role not only in the cases of infertility or subfertility but also in the population of fertile males. In light of this association, under physiological conditions, the Fe level in semen seems to be more closely related to fertility potential than the levels of other examined metals.

To counteract the destructive effects of ROS, seminal plasma has an antioxidant defense system [34] that is a source of antioxidant enzymes, such as SOD, CAT, and GPx. SOD converts superoxide anions to hydrogen peroxide which is, in turn, utilized by CAT and GPx. GPx activity is related to the level of reduced glutathione (GSH). GSH is a major thiol antioxidant in the human body that plays a central role in the defense against oxidative stress by being oxidized to glutathione disulfide (GSSG). To restore GSH content, GSSG is reduced by GR, which requires NADPH provided by G6PD. The second GSH-dependent enzyme is GST. The main role of GST is the utilization of xenobiotics [35, 36]. Apart from GSH, seminal plasma is rich in other nonenzymatic antioxidants, such as ascorbate, urate, alpha-tocopherol, and pyruvate [1]. In the present study, the activities of SOD, CAT, and G6PD were higher in the EX group than in the ME group. This observation suggests higher effectiveness of the enzymatic antioxidant defense in the group of males with better sperm quality. Consistently, several authors suggest that lower activities of SOD and CAT may be associated with decreased fertility. Moreover, activities of these enzymes have been shown to correlate positively with sperm quality markers, such as motility, sperm count, and sperm volume [34, 37–39]. However, the associations between the antioxidant defense system parameters and oxidative stress are not conclusive because there are also studies reporting inconsistent results. Therefore, it has been proposed that each studied pathology connected with poor semen quality, such as varicocele or genitourinary infections, may be related to a definite pattern of markers of oxidative stress and nonenzymatic antioxidant compounds [40].

The major source of ROS in seminal plasma is sperm metabolism [41]. Therefore, the higher sperm motility observed in the EX group than that in the ME group should be associated with elevated oxidative stress due to the higher energy demand of motile spermatozoa. Nevertheless, the levels of measured oxidative stress biomarkers, such as MDA, LPS, and TOS, as well as TAC value and the concentration of thiol groups that reflect antioxidative potential, did not differ between the examined groups. This observation also suggests that the EX group possesses a more effective antioxidant defense system. Surprisingly, only the level of alpha-tocopherol, a powerful nonenzymatic antioxidant, was significantly decreased in the EX group compared to the ME group. Studies generally support the beneficial role of this form of vitamin E in maintenance of male fertility potential [42–44]. Therefore, the results in the present study need to be verified.

Proinflammatory cytokines, such as IL-1*β*, IL-6, IL-8, IL-12, and TNF-*α*, are believed to contribute to poor sperm quality by inducing oxidative stress and lipid peroxidation [45]. However, in the present study, levels of cytokines, including the proinflammatory ones, did not differ between the examined groups. This suggests that semen of fertile males is rather homogenous with respect to immune system parameters.

## 5. Conclusions

The seminal plasma pH is not related to the semen quality of fertile males. Higher fertility potential estimated based on standard semen parameters in fertile males is associated with lower levels of Fe and higher activities of some antioxidant enzymes due to lower oxidative stress intensity.

## Figures and Tables

**Table 1:** Semen parameters in the study population (group ME: mediocre semen morphology and group EX: excellent semen morphology). *p* value—Student's *t*-test.

	ME group *n* = 52		EX group *n* = 51		*p* value	Relative change
	Mean	SD	Mean	SD		
Age	33.9	6.02	32.4	5.64	0.492	−4%
Volume (ml)	3.44	1.44	4.04	1.97	0.038	17%
pH	7.58	0.08	7.55	0.06	0.080	—
Sperm cell count in 1 ml (mln/ml)	73.0	66.6	84.8	50.4	0.011	16%
Total sperm cell count (mln)	229	198	312	173	0.001	36%
Motile sperm cells after 1 hour (%)	54.1	7.80	62.3	9.39	<0.001	15%
Progressively motile sperm cells after 1 hour (%)	22.9	9.1	29.7	9.55	<0.001	30%
Motile spermatozoa after 24 hours (%)	14.7	13.1	23.3	17.1	0.005	58%
Progressively motile spermatozoa after 24 hours (%)	4.60	5.85	8.38	9.20	0.014	82%
Normal morphology (%)	49.4	8.11	54.0	7.22	0.005	9%

*p* < 0.05.

**Table 2:** Semen parameters in the study population (group ME: mediocre semen morphology and group EX: excellent semen morphology). *p* value—Student's *t*-test.

	ME group		EX group		*p* value	Relative change
	Mean	SD	Mean	SD		
Phosphates concentration (mg/dl)	97.4	67.8	66.7	33.9	0.033	−31%
Magnesium concentration (mg/dl)	6.15	3.37	5.72	4.16	0.272	−7%
Calcium concentration (mg/dl)	30.2	10.25	29.4	11.5	0.605	−3%
Iron concentration (*μ*g/dl)	4.74	3.68	3.55	3.60	*0.064*	−25%
Zinc concentration (mg/l)	123	54.8	134	59.1	0.650	8%
Copper concentration (*μ*g/dl)	34.6	46.3	36.6	45.1	0.351	6%
Selenium concentration (*μ*g/l)	26.0	8.89	28.8	16.67	0.849	10%
Lead concentration in seminal plasma (*μ*g/dl)	0.99	0.49	1.22	0.57	0.116	23%

*p* < 0.05.

**Table 3:** The levels or activity of malondialdehyde (MDA), lipofuscin, total oxidant status (TOS), superoxide dismutase (SOD), Mn-SOD, CuZn-SOD, thiol groups, catalase (CAT), glutathione reductase (GR), glutathione peroxidase (GPx), glutathione-S-transferase (GST), glucose-6-phosphate dehydrogenase (G6PD), total antioxidant capacity (TAC), and *α*-tocopherol in the study population (group ME: mediocre semen morphology and group EX: excellent semen morphology). *p* value—comparison between the ME and EX groups using Student's *t*-test.

	ME group		EX group		*p* value	Relative change
	Mean	SD	Mean	SD	Mean	SD
MDA concentration (*μ*mol/l)	2.32	0.81	2.51	1.13	0.474	8%
Lipofuscin concentration (RF)	3.81	1.50	4.02	1.65	0.975	5%
TOS (*μ*mol/l)	11.00	14.23	11.0	13.7	0.697	0%
SOD activity (NU/mg protein)	4.09	1.59	4.62	1.50	*0.059*	13%
Mn-SOD activity (NU/mg protein)	0.83	1.06	0.88	0.93	0.358	6%
CuZn-SOD activity (NU/mg protein)	3.12	1.46	3.55	1.30	0.050	14%
Thiol group concentration (*μ*mol/g protein)	4.73	2.46	5.43	2.70	0.098	15%
CAT activity (U/g protein)	11.9	7.73	19.3	20.6	0.030	63%
GR activity (U/g protein)	1.64	1.58	1.85	2.09	0.668	13%
GPx activity (U/g protein)	3.13	4.71	1.62	2.91	0.273	−48%
GST activity (mU/g protein)	83.3	60.9	84.8	55.1	0.732	2%
G6PD activity (U/g protein)	1.22	1.09	1.70	1.23	0.049	39%
TAC (mmol/l)	1.38	0.27	1.29	0.22	0.199	−7%
*α*-Tocopherol concentration (*μ*g/ml)	10.60	3.82	7.18	3.78	<0.001	−32%

*p* < 0.05.

**Table 4:** The levels of interleukin 1*β* (IL-1*β*), interleukin 2 (IL-2), interleukin 4 (IL-4), interleukin 5 (IL-5), interleukin 6 (IL-6), interleukin 7 (IL-7), interleukin 8 (IL-8), interleukin 10 (IL-10), interleukin 12 (p70), interleukin 13 (IL-13), interleukin 17 (IL-17), granulocyte colony-stimulating factor (G-CSF), granulocyte-macrophage colony-stimulating factor (GM-CSF), interferon gamma (IFN-*γ*), monocyte chemoattractant protein-1 (MCP-1), macrophage inflammatory protein 1-*α* (MIP-1*α*), and tumor necrosis factor *α* (TNF-*α*) in seminal plasma of the examined population. *p* value comparison between values obtained in the ME group: mediocre semen morphology and EX group: excellent semen morphology using Mann-Whitney *U* test.

	ME group		EX group		*p* value
	Median	IQR	Median	IQR	
IL-1*β* (pg/ml)	1.29	2.60	1.07	2.61	0.450
IL-2 (pg/ml)	0.00	1.01	0.02	3.62	0.284
IL-4 (pg/ml)	0.09	0.02	0.11	0.17	0.494
IL-5 (pg/ml)	22.3	56.6	49.8	118	0.604
IL-6 (pg/ml)	12.0	17.8	7.13	11.6	0.238
IL-7 (pg/ml)	508	444	606	409	0.116
IL-8 (pg/ml)	179	190	146	156	0.752
IL-10 (pg/ml)	1.37	1.12	1.39	2.27	0.770
IL-12 (p70) (pg/ml)	2.32	3.93	2.80	3.53	0.913
IL-13 (pg/ml)	0.35	1.01	0.42	0.46	0.688
IL-17 (pg/ml)	6.50	8.80	8.07	8.79	0.971
G-CSF (pg/ml)	17.9	29.8	16.1	27.4	0.971
GM-CSF (pg/ml)	188	65.88	196	53.2	0.618
IFN-*γ* (pg/ml)	57.1	71.3	40.8	93.0	0.643
MCP-1 (MCAF) (pg/ml)	1139	1880	903	1038	0.415
MIP-1b (pg/ml)	46.8	34.9	57.6	35.0	0.458
TNF-*α* (pg/ml)	3.52	2.82	4.76	4.03	0.569

IQR: interquartile range; *p* < 0.05.

## Data Availability

The data used to support the findings of this study are available from the corresponding author upon request.
